# Hypertension in Women

**DOI:** 10.3389/fcvm.2022.905504

**Published:** 2022-06-03

**Authors:** Tatjana Tasić, Marijana Tadić, Maja Lozić

**Affiliations:** ^1^School of Dental Medicine, University of Belgrade, Belgrade, Serbia; ^2^Clinic for Internal Medicine II, Cardiology Department, University Clinic of Ulm, Ulm, Germany; ^3^Centre for Discovery Brain Sciences, University of Edinburgh, Edinburgh, United Kingdom

**Keywords:** postmenopausal hypertension, pregnancy induced hypertension, antihypertensive treatment, sex hormones, sex differences

## Abstract

Hypertension is one of the main causes of morbidity and mortality in the human population. Nevertheless, the intricate network of pathophysiological mechanisms that lead to the development of hypertension in women still awaits to be fully understood. From young age to maturity and senescence, the female body transits through different stages, each of them characterized with specific physiological features and disposition to particular pathological conditions, and that is exactly what makes the understanding of the genesis and adequate treatment of hypertension in women so challenging. Clinical and experimental findings emphasize the role of sex hormones, autonomic nervous system, renin-angiotensin-aldosterone system and arterial stiffness in the development of chronically elevated blood pressure in females. The purpose of this review is to briefly summarize the knowledge of the mechanisms and treatment of hypertension in women.

## Introduction

Hypertension is a major risk factor for cardiovascular events and one of the leading causes of morbidity and mortality worldwide. It is known that adult women in the reproductive period have slightly lower blood pressure than men of the same age. Many clinical studies pointed out that men have a higher prevalence of hypertension than premenopausal women, but postmenopausal women with hypertension are more susceptible to development of isolated systolic hypertension, with less efficient therapeutic response compared to the age-matched men (1). According to the National Health and Nutrition Examination Survey (NHANES) conducted on 9,623 participants of the U.S. adult population, the prevalence of hypertension among women older than 75 years was 78% (2). Also, it has been noticed that elderly women are under high cardiovascular event risk, probably due to impaired dipping pattern (3). Impaired dipping pattern is a predictor of cardiovascular events with prognostically unfavorable outcomes (4–7) and may be defined either as a decrease in blood pressure during sleep that exceeds 20% of blood pressure values measured during wake time, or increase in blood pressure during sleep. Hypertension that occurs in women seems to be more sensitive to salt loading and it is more frequently associated with the metabolic syndrome than it is in men. These specific features of hypertension in women that were somewhat overlooked in the past might lead to the diminished responsiveness to the existing antihypertensive therapy (8, 9). Also, it is worth mentioning that women, more likely than men, will develop side effects on antihypertensive drugs (9). All of this suggests that more comprehensive, sex-oriented approach to the treatment of hypertension should be considered in future (9, 10). This review will try to elucidate the mechanisms, outcome and treatment of hypertension in women.

## Premenopausal Hypertension

Before reaching menopause at the age of 45 to 55 years, women have slightly lower blood pressure levels and also lower chance to develop hypertension than the age-matched men. A large body of evidence indicates that sex hormones are responsible for the sex differences in the regulation of blood pressure (11). It has been shown that sex hormones affect systems that are considered to play an important part in the development of hypertension, such as renin angiotensin aldosterone system, endothelin, nitric oxide (NO) system and immune system. Estrogen is believed to have beneficial effects on cardiovascular system and it possibly exerts its protective role in women in reproductive age through enhancement of NO-mediated vasodilatation and modulation of action of powerful vasoconstrictor endothelin-1 action (11). Expression of endothelin-1 receptor ET_1A_ is significantly higher in hypertensive male rats than it is in the hypertensive females of the same age, and this difference in receptor density is assigned to the opposing effects of androgens and estrogen on endothelin receptor expression (11). In addition, the differences in endothelin-1 levels were registered in human population, with men having higher levels of endothelin-1 than women (11). Also, brain estrogen has modulatory role in control of the cardiovascular system and this matter will be elaborated later in the text.

In general, causes, outcome and treatment of the primary (essential) hypertension do not differ significantly between young adult women and men of the same age. Although, some particular clinical features distinguish hypertension in premenopausal period, for instance increased cardiac index and left ventricular ejection time, higher values of resting heart rate, but decreased plasma renin levels, blood volume and total peripheral vascular resistance (12). In contrast to elderly women, who are predominantly diagnosed with essential hypertension, hypertension in premenopausal women is usually caused by another condition or a disease. Major causes of the secondary hypertension in younger women are obesity, polycystic ovarian syndrome, obstructive sleep apnea, coarcatation of aorta, Turner syndrome, autoimmune diseases, endocrine disorders (hyperaldosteronism, hypothyroidism, hyperthyroidism, hyperparathyroidism, pheochromocytoma, diabetes mellitus), kidney diseases and drug usage (e.g., corticosteroids, hormonal contraceptives, etc.) (13–15). Use of oral contraceptives has been linked to the higher incidence of hypertension in the population of women in the reproductive age (16, 17), and the existing evidence shows that estrogen component in particular leads to the activation of renin angiotensin aldosteron system, potentiates sodium retention and plasma volume expansion (18). Therefore, contraceptive pills containing only progestin or combined oral contraceptives comprising of drospirenone (progestin with antimineralcorticoid activity) and low dose of the estradiol are recommended for women with the risk of developing hypertension (18, 19).

## Postmenopausal Hypertension

Changes in heart morphology, function and compliance of arterial blood vessels have all been recognized and described in postmenopausal hypertension. Postmenopausal women have higher incidence of left ventricular hypertrophy and they are at greater risk to developed diastolic dysfunction compared to young adult women (20). Groban et al. (21) showed that female Lewis rats that underwent bilateral ovariectomy developed progressive diastolic dysfunction. Mori et al. (22) reported that ovariectomized hypertensive rats had cardiac hypertrophy, myocardial fibroses and diastolic dysfunction, but 17β-estradiol replacement treatment prevented these changes. The isolated systolic hypertension in postmenopausal women is related to aortic stiffness, probably caused by smooth muscle cells proliferation, collagen accumulation and increased levels of vasoconstrictor molecules in the blood vessel wall due to the lack of estrogen protective effect (20). Maiello et al. (23) showed that global pulse wave velocity, a reliable indicator of aortic stiffness, is highly increased in postmenopausal women with hypertension.

### Role of Estrogen in Postmenopausal Hypertension

The complex regulatory pattern and multiple factors are associated with postmenopausal hypertension. Estrogen deprivation might be one of the leading causes of hypertension in postmenopausal women. Previously performed studies had shown that estradiol reduces blood pressure in rat animal models with genetic or induced hypertension (24). It has been reported that aged spontaneously hypertensive female rats, used as an animal model of postmenopausal hypertension, have reduced serum levels of estradiol and increased levels of androgens after cessation of regular cycling (25). Furthermore, mice treated with 4-vinylcyclohexene diepoxide (VCD), which causes ovarian failure, achieved increase in blood pressure compared to control animals and estradiol treatment had protective role on VCD mice (26). Epidemiological and experimental data imply that estrogen could cause vasodilatation due to effects on the renin angiotensin system, NO system, endothelin, and immune system (8, 27, 28). The renin angiotensin aldosteron system is important for hydromineral balance and it has a well-established role in the genesis of hypertension. Estradiol increases angiotensinogen gene expression due to modulation of gene promoter, reduces gene expression of angiotensin AT_1_ receptors, suppresses angiotensin converting enzyme and plasma renin activity (3). Further, estradiol stimulates production of endothelial NO synthase that provides NO, important factor for vasodilatation. The lack of estradiol can also affect NO bioavailability due to reduced activity of superoxide dismutase (25, 28). Best et al. (29) reported that treatment with 17β-estradiol reduced plasma nitric oxide and endothelin-1 in postmenopausal women. The elevated level of powerful vasoconstrictor agent endothelin is registered in postmenopausal women and postcycling spontaneously hypertensive rats. It is found that estrogens inhibit endothelin synthesis and decrease endothelin ET_1A_ receptors expression (11, 28). Noteworthy, preclinical studies showed that angiotensin II stimulates endothelin production, and postmenopause is characterized by enhanced activity of renin angiotensin system (28). Furthermore, the role of estrogen as immune system modulator could not be neglected in postmenopausal hypertension. Estrogen might have antiinflammatory role in immune response by affecting humoral immune system and T cellular immune system (11, 30).

### One Look at “Overlooked” Hormones: Progesterone and Androgens in Postmenopausal Hypertension

Menopause, as well as ovariectomy, does not result only in the loss of estrogen. Several studies have shown that precipitous decline in progesterone levels might be, at least in part, associated with the occurrence of arterial hypertension in postmenopausal women. Barbagallo et al. (31) demonstrated that progesterone acts as a vasoactive hormone, preventing noradrenaline-induced vasoconstriction by acting directly on vascular smooth muscle cells. These results further confirm previous *in vitro* findings of Jiang et al. (32) that revealed endothelium-independent vasodilatory effect of progesterone on rabbit coronary arteries. In a pilot study conducted in humans, natural progesterone lowered diastolic and mean blood pressure values in both men and postmenopausal women (33), while Seely et al. (34) noticed that intravaginal progesterone in combination with transdermal estrogen lowers night time blood pressure in healthy postmenopausal women. In postmenopausal women with arterial stiffness and grade 1 hypertension, introduction of progesterone to the ongoing estrogen-replacing treatment did not adversely affect positive cardiovascular effects achieved by estrogen (35). According to another study, micronized progesterone in combination with conjugated equine estrogen can induce even larger decrease in systolic blood pressure in hypertensive postmenopausal women than estrogen alone (36). At the first glance, these novel findings seem to contradict the results of studies published in the past (37) that offered evidence of dose-dependent hypertensive effect of progestogens in oral contraceptives. The reason for this discrepancy possibly lies in the fact that, unlike natural progesterone, synthetic progestins in oral contraceptives and hormone replacement therapy possess androgenic activity (38).

Androgens have been implied to participate in the development of cardiovascular disorders in postmenopausal women, although their exact role in the etiopathogenesis of postmenopausal hypertension still remains ambiguous. Given the fact that in the reproductive period men have higher risk of developing cardiovascular disorders in comparison to age-matched women, it has been assumed that androgen steroids have detrimental impact on cardiovascular system (39). This assumption seemed to be further substantiated with the results showing that women with polycystic ovary syndrome, the condition characterized by increased level of plasma androgens, were found to be at greater risk of developing hypertension (14). In a basic study conducted in normotensive rats of both sexes, daily administration of dihydrotestosterone (androgen steroid derived from testosterone) for 2 weeks induced significant increase in systolic blood pressure, possibly through mechanisms that promote the production of cytochrome P450-derived metabolites of arachidonic acid with potent vasoconstrictor properties (e.g., 20-Hydroxyeicosatetraenoic acid) (40). Androgen steroid propensity to increase blood pressure and potentiate adrenergic vasoconstriction of aorta was also noticed after addition of testosterone to the estrogen replacement therapy in ovariectomized spontaneously hypertensive female rats (41). Other mechanisms that might be involved in androgen-induced hypertension are stimulation of renin angiotensin system, vasoconstriction caused by potentiation of endothelin activity and oxidative stress (25, 28).

Nevertheless, there is a considerable amount of published data showing that it is not hyperandrogenemia, but androgen deficiency that is associated with the development of cardiovascular disorders (42–45). In the Rancho-Bernardo study, Laughlin et al. (46) showed that when compared to men with normal levels of testosterone, older men with low levels of total testosterone had 40% higher risk of death over the following 20 years, independently of age, body weight or life style. In postmenopausal women, low levels of dehydroepiandrosterone sulfate, androgen and precursor of steroid hormones, have been associated with higher cardiovascular mortality and with the increase in all-cause mortality (45, 47).

### Role of Autonomic Nervous System in Postmenopausal Hypertension

Development of postmenopausal hypertension may be linked to the age-related alterations in autonomic nervous system (48).

Sympathetic nerve activity (SNA), which can be regarded as an output of central sympathetic outflow, progressively increases with age, and in menopausal women, this increase in SNA appears to be significantly steeper than in men of the same age (49, 50). Denervation of renal sympathetic nerve in spontaneously hypertensive female rats reduced blood pressure more efficiently in old dams than in young counterparts (51). Barnes et al. (52) reported that ganglionic blockade caused a larger drop of blood pressure in postmenopausal women compared to young women. The greater vasoconstrictor response following the administration of noradrenaline accompanied with blunted beta receptor-mediated vasodilatory protective effect is also noticed in postmenopausal women when compared to young women (53).

There are two crucial changes in autonomic regulation that arise during menopause that are able to facilitate the development of hypertension–increase in central sympathetic outflow and enhancement of adrenergic sensitivity in peripheral blood vessels (48). Here, we will focus only on the changes in the central autonomic control in postmenopausal women.

The major centers in the brain that adjust sympathetic outflow to the periphery are hypothalamic paraventricular nucleus (PVN), nucleus tractus solitarius (NTS), rostral ventrolateral medulla (RVLM) and central ventrolateral medulla (CVLM) (54, 55). Sympathetic tone is generated in RVLM which integrates inputs from other parts of the brain (PVN, NTS, CVLM), and from RVLM nerve impulses are transmitted to sympathetic preganglionic neurons in intermediolateral column (ILC) of the thoracolumbar part of the spinal cord. From ILC sympathetic nerves further transmit nerve impulses to effector organs (heart, kidney, adrenal gland and blood vessels) (55). Parasympathetic tone is generated in NTS. The afferent nerve fibers transmit nerve signals from baroreceptors in the carotid sinus and aortic arch to the NTS in brainstem. From NTS, these signals are further conveyed to the nucleus ambiguous and dorsal motor nucleus of vagus and via parasympathetic nerve fibers they reach the heart (55).

Estrogen receptors (ERα and ER_β_) are found in all of the aforementioned brain centers involved in the central control of cardiovascular system (56, 57) and according to the number of studies, their activation consequently leads to the sympathoinhibition.

In one of these studies (58), sympathoinhibitory effect of estrogen was demonstrated in the group of postmenopausal women, who experienced the decrease in muscle sympathetic activity after transdermal application of estrogen. Experiments in laboratory animals also suggest that estrogen wields regulatory influence on the autonomic nervous system during and after menopause. Intracerebroventricular administration of estrogen attenuated hypertension in female rats that were subjected to ovariectomy (56), while injections of estrogen into the NTS, RVLM, nucleus ambiguous and several other hindbrain nuclei, decreased mean blood pressure and renal sympathetic nerve activity and enhanced cardiac baroreflex in ovariectomized female rats (59). Similar effect on blood pressure was noticed after estrogen application into one of the major integrative autonomic centers - PVN. The activation of ER_β_ receptors in the PVN leads to decline in production of reactive oxygen species and activates the neural nitric oxide synthetase, promoting the production of nitric oxide (48, 56). Exciting results from the recent study (57) reveal that the administration of ER_β_ receptor agonist in the PVN of perimenopausal female mice prevents the increase in blood pressure in the angiotensin II-induced neurogenic hypertension. The importance of ER_β_ receptor signaling in the modulation of central autonomic control is further confirmed by the findings that demonstrate that estradiol inhibits pressor effects originating from RVLM *via* activation of ER_β_/PI3K/Akt signaling pathway (60).

### Role of Immune System in Hypertension- Sex Differences

Recent studies have shown possible role of immune system in the development of hypertension (61–63). It has been hypothesized that dysregulation of the T cells immune response may contribute to hypertension (64). Results obtained from both hypertensive animals and humans registered decline of blood pressure after administration of immunosuppressant mycphenolate mofetil, indicating the possible link between inflammation and the development of hypertension (61, 62).

Studies on recombinant activating gene-1 defficient (Rag^−/−^) mice that lack both T and B cells, showed that T cells in females have lower proinflammatory and prohypertensive potential in respect to T cells of males (62, 65). Furthermore, spontaneously hypertensive males have increased number of pro-inflammatory and pro-hypertensive T helper 17 (Th17) cells in kidneys, while increased count of T regulatory cells has been found in kidneys of female hypertensive rats (61). Abovementioned difference in T-cell subpopulations found in kidneys of female and male rats may be a consequence of different cytokine profile found between sexes. Tipton and Sullivan (61) reported elevated levels of transforming growth factor-β, tumor necrosis factor-α, interleukin-10 in female spontaneously hypertensive rats, in contrast to higher levels of interleukin-6 and interleukin-17 found in renal cells. Furthermore, different expression and activation of Toll-like receptors present on immune cells could be responsible for more pronounced proinflammatory response and the development of hypertension in males (63).

## Pregnancy Induced Hypertension

There are different types of pregnancy induced hypertension: preeclampsia, chronic hypertension with superimposed preeclampsia, gestational hypertension and chronic hypertension (66). Preeclampsia consists of hypertension that arises 20 weeks after gestation combined with proteinuria (66). Chronic hypertension with superimposed preeclampsia sublimes preexisting hypertension with other hallmarks of preeclampsia (66). Gestational hypertension develops 20 weeks after gestation and it is maintained only up to 12 weeks after parturition (66). Chronic hypertension may be defined as elevated blood pressure that appears before week 20 of gestation or as hypertension diagnosed for the first time 20 weeks after gestation and that continues to persists 12 weeks after the labor (66). Previous epidemiological studies showed that pregnancy-induced hypertension could cause consequences on either maternal or fetal health. During normal pregnancy mother‘s organism goes through numerous physiological changes necessary to adapt to the fetus. The cardiac output increases due to the increment in heart rate and stroke volume. The arterial blood pressure rises during pregnancy, but it decreases near the time of the term, ultimately reaching pregestational values. Due to diminished response of peripheral blood vessels to angiotensin II, peripheral vascular resistance decreases during the normal pregnancy. With the approaching labor, circulatory volume increases by 40% in order to provide proper fetal supply of oxygen and nutrients, and to prepare mother for blood loss during labor. Many of the hemodynamic adaptations during pregnancy may be assigned to relaxin. This polypeptide is synthetized by corpus luteum and it is mainly responsible for vascular remodeling and vasodilatation, which is predominantly achieved by the stimulation of NO system and alteration in the expression of endothelin ET_1B_ receptors (67). Beside aforementioned hemodynamic changes, pregnancy is also characterized by the increase in renal blood flow, glomerular filtration rate, activity of the renin angiotensin aldosterone system and enhanced activity of the sympathetic nervous system.

Preeclampsia is the major risk factor that can lead to the vast number of life threatening complications like preterm delivery, placental abruption, ischemic stroke, disseminated intravascular coagulation, renal failure, Elevated Liver Enzymes and Low Platelets syndrome (HELLP), fetal growth restriction and fetal intrauterine death (66). Although the exact mechanisms underlying the development of preeclampsia still need to be elucidated, it is assumed that the factors produced by placenta, altered immune system and genetic factors have a role in the of genesis preeclampsia. Healthy pregnancy is accompanied by fetal cytotrophoblast invasion of mother's spiral arteries in order to supply fetus with oxygen and other nutrients. Studies showed that placentas of women with preeclampsia have damaged uteroplacental blood flow based on no adequate modification of spiral arteries and their increased resistance (68). It is hypothesized that endothelial dysfunction caused by disbalance among molecules responsible for angiogenesis contribute to the occurrence of preeclampsia. Placental failure to adequately respond in preeclampsia is linked with its production of solubile fms-like tyrosine kinase 1 and soluble Endoglin (69). These factors are receptors of vascular endothelial growth factor, placental growth factor and transforming growth factor β, which are the main molecules in the regulation and adaptation of maternal-fetal blood flow. The role of immune system in pathogenesis of preeclampsia cannot be neglected. Changes in placenta implementation is probably responsible for the impaired immune response and development of preeclampsia. Women with preeclampsia have decreased regulatory T cells and overexpressed different types of Toll-like receptors in respect to normotensive pregnant women (61, 63).

## Antihypertension Therapy In Women

Except during pregnancy, available guidelines for the management of hypertension do not offer different therapeutic approach to women and men. Firstly, lifestyle modification is highly recommended for blood pressure control in women (8, 12). Hypertension in postmenopausal women tends to be salt sensitive, so reduction of sodium intake could have benefit on blood pressure decrease. Further, weight lost, increased physical activity, decreased alcohol consumption, diet based on vegetables and fruits with elimination of fat dairy products have their role in hypertension management (70). Some studies pointed out that diet based on phytoestrogens might have protective effects on cardiovascular system (20). Many studies showed the prevalence and effects of the antihypertensive drugs (beta blockers, renin angiotensin aldosterone system inhibitors, calcium channel blockers, diuretics) usage in women and men (10). Briefly, men were more frequently treated with calcium channel blockers, angiotensin converting enzyme inhibitors, but were less frequently treated with diuretics, beta blockers and angiotensin II receptor blockers in respect to women. The sex difference response to pharmacotherapy is probably related to different metabolism of drugs and diseases linked to hypertension that are more common in women than men. Thus, hypertension in postmenopausal women is often associated with metabolic syndrome and autoimmune diseases which lead to less therapy efficacy and higher risk of cardiovascular complications (9). The absorption, distribution, metabolism and excretion of antihypertensive drugs are different between women and men probably due to influence of sex hormones on the absorption transporters (P-glycoprotein), distribution volume, activity of cytochrome P450 (CYPs) and renal clearance (10, 13). For example, women drug metabolism is altered in respect to men due to increased activity of CYP3A4, CYP2A6, CYP2B6 and decreased activity of CYP1A2, CYP2E1 and P-glycoprotein (10, 13). It is reported that side effects of some antihypertensive treatment are more common in women than men (12). Hence, women treated with angiotensin converting enzyme inhibitors have induced cough more frequently than men, also treatment with diuretics is associated with hyponatremia and hypokalemia in women (3, 19). Opposite to that, postmenopausal women have decreased risk of bone fracture if diuretics are recommended treatment (3). Furthermore, women are more prone to peripheral edema development during calcium channel blocker usage and minoxidil induced hirsutism, in respect to men (3, 19). During pregnancy some of antihypertensive drugs are not recommended in order to escape fetal toxicity and malformation (15, 18). Angiotensin converting enzyme inhibitors, angiotensin II receptor blockers and direct renin inhibitors could cause fetal growth restriction and death (15). American College of Cardiology/American Heart Hypertension Guideline recommended methyldopa, nifedipine and labetalol for hypertension treatment during pregnancy (13).

## Conclusion

There are still many controversies and unsolved questions in the attempts to comprehend the mechanisms that lie behind pathophysiology of hypertension in women, which surpass our need to merely satisfy scientific curiosity. With hypertension as one of the major risk factors for cardiovascular events in elderly women, and preeclampsia still being the leading cause of maternal death in the countries of developed world, better understanding of hypertension in women is needed for reevaluation of our approach to the treatment of this condition.

## Author Contributions

All authors listed have made a substantial, direct, and intellectual contribution to the work and approved it for publication. All authors contributed to the article and approved the submitted version.

## Conflict of Interest

The authors declare that the research was conducted in the absence of any commercial or financial relationships that could be construed as a potential conflict of interest.

## Publisher's Note

All claims expressed in this article are solely those of the authors and do not necessarily represent those of their affiliated organizations, or those of the publisher, the editors and the reviewers. Any product that may be evaluated in this article, or claim that may be made by its manufacturer, is not guaranteed or endorsed by the publisher.

## Abbreviations

ACE: angiotension converting enzyme
AT: angiotensin
ET: endothelin
NO: nitric oxide
VCD: vinylcyclohexene
20-HETE-20: Hydroxyeicosatetraenoic acid
SNA: sympathetic nerve activity
PVN: paraventricular nucleus
NTS: nucleus tractus solitaries
RVLM: rostral ventrolateral medulla
CVLM: central ventrolateral medulla
HELLP, Hemolysis: Elevated Liver enzymes and Low Platelets
CYPs: cytochrome P450.
